# Process and kinetics of azo dye decolourization in bioelectrochemical systems: effect of several key factors

**DOI:** 10.1038/srep27243

**Published:** 2016-06-07

**Authors:** Hou-Yun Yang, Chuan-Shu He, Lei Li, Jie Zhang, Jin-You Shen, Yang Mu, Han-Qing Yu

**Affiliations:** 1 CAS Key Laboratory of Urban Pollutant Conversion, Collaborative Innovation Centre of Suzhou Nano Science and Technology, Department of Chemistry, University of Science and Technology of China, Hefei, China; 2 Jiangsu Key Laboratory of Chemical Pollution Control and Resources Reuse, School of Environmental and Biological Engineering, Nanjing University of Science and Technology, Nanjing 210094, Jiangsu Province, China

## Abstract

This study explored the influence of several key factors on the process and kinetics of azo dye decolourization in bioelectrochemical systems (BESs), including cathode potential, dissolved oxygen (DO) concentration of catholyte and biofilm formed on the cathode. The results show that azo dye methyl orange (MO) decolourization in the BES could be well described with the pseudo first-order kinetics. The MO decolourization efficiency increased from 0 to 94.90 ± 0.01% and correspondingly the reaction rate constant increased from 0 to 0.503 ± 0.001 h^−1^ with the decrease in cathodic electrode potential from −0.2 to −0.8 V vs Ag/AgCl. On the contrary, DO concentration of the catholyte had a negative impact on MO decolourization in the BES. When DO concentration increased from zero to 5.80 mg L^−1^, the MO decolourization efficiency decreased from 87.19 ± 4.73% to 27.77 ± 0.06% and correspondingly the reaction rate constant reduced from 0.207 ± 0.042 to 0.033 ± 0.007 h^−1^. Additionally, the results suggest that the biofilm formed on the cathode could led to an adverse rather than a positive effect on azo dye decolourization in the BES in terms of efficiency and kinetics.

Azo dyes in the wastewater not only pose a severe threat to the environment and lives but also affect the transparency and appearance of natural water bodies because of their toxicity and bioaccumulation^1^. Many physico-chemical technologies, such as adsorption, coagulation, flocculation and advanced oxidation, have been developed for azo dyes removal from wastewater^2^, but are suffering different shortages including complex operational options, high cost, and producing large amount of sludge which need further overcome. Biological treatment was proposed as an alternative approach to improve the economic efficiency in azo dye wastewaters treatment, including bacteria^3^, fungi^4^ and enzymes^5^. However, those methods are also facing several problems such as high operating and maintenance costs, difficulties in the practical application, and enzyme instability.

Bioelectrochemical systems (BESs), such as microbial fuel cells (MFCs) and microbial electrolysis cells (MECs) have been explored extensively for their innovative features and environmental benefits^6^,^7^. Since energy generation is not always the aim of the technology, the versatility of BESs has been demonstrated by numerous valuable oxidation and reduction reactions^8^. For instance, BESs have been applied to remove organic and inorganic contaminants such as acetate^9^,^10^, cellulose^11^, and sulphide^12^,^13^ from wastewater at the anode chamber. On the other hand, BESs could produce hydrogen^9^,^14^, reduce nitrate and nitro-aromatics^15^,^16^,^17^, perchlorate, hexavalent chromium, halogenated aromatics and chloramphenicols^18^ at the cathode chamber.

In recent years, BES technology has drawn increasing interest in the decolourization of azo dyes from wastewater^19^,^20^,^21^. Mu *et al.* constructed a BES, where the process was driven by microbial oxidation of acetate at the anode, to break the azo bonds of azo dye using proton and electron in the cathode chamber^22^. Cui *et al.*, Kong *et al.* and Savizi *et al.* focused on the azo dye decolourization in the BESs with dual microbial or enzymatic cathode^23^,^24^,^25^. Kong *et al.* modified a sleeve-type configuration with an inner anode chamber and an outer cathode chamber to decolourize azo dye^20^. Moreover, several practical applications for azo dyes treatment were preconceived for the development of modified BES configurations including the membrane-free double-chamber BES^26^, the integrated biocatalyzed electrolysis and bio-contact oxidation reactor^27^, and the assembled pilot-scale baffled reactor with the biocatalyzed electrolysis system^28^ to satisfy the scale employment.

Previous studies showed that cathode potential could substantially influence azo dye decolourization in BESs. Mu *et al.* reported that the decolourization of azo dye acid orange 7 was significantly enhanced by the controlled cathodic potential in the range of −0.35 to −0.55 V vs SHE (Standard Hydrogen Electrode) in the BES^22^. The similar results were also observed by Cui *et al.* and Sun *et al.* for azo dye removal in BESs^24^,^29^, respectively. On the other hand, development of biocathode for enhanced removal of nitroaromatics, halogenated aromatics and azo dyes has obtained more and more interest in the BES^23^,^25^,^30^,^31^,^32^,^33^,^34^,^35^, but the enrichment of not only specific pollutant-reducing and but also electrochemically active consortium on the cathode of the BES is much difficult and moreover the process is also time-consuming^36^. In such conditions, some other types of microorganisms rather than the electrochemically active bacteria would potentially be enriched on the cathode if co-substrate is inevitable in the wastewater, which might led to an undesirable consequence for azo dye removal in the BES. Additionally, oxygen might be present in the real dye wastewater, but no reports were found to investigate the effect of dissolved oxygen (DO) on azo dye removal in the BES. Since oxygen is an attractive oxidant because of its thermodynamic high redox potential^37^, it could speculate that oxygen, if present in the wastewater, may compete for electrons with azo dye and thus affect decolourization performance in the BES. In summary, the information about the influence of these key factors on azo dye decolourzation in the BES is still limited especially in terms of kinetics.

Therefore, this study aimed at evaluating the influence of several key operational parameters on azo dye decolourization in BESs, including cathode potential, DO concentration of catholyte and biofilm formed on the cathode. Methyl orange (MO) was used as a model azo dye because it is widely presented in dyeing wastewater^38^. Both MO decolourization and formation of its reductive products were investigated under different conditions in the BESs. Moreover, the MO decolourization kinetics in the BESs was also elucidated with a pseudo-first order reaction model in this study.

## Results and Discussion

### Typical MO decolourization in the BES

**Figure 1:**
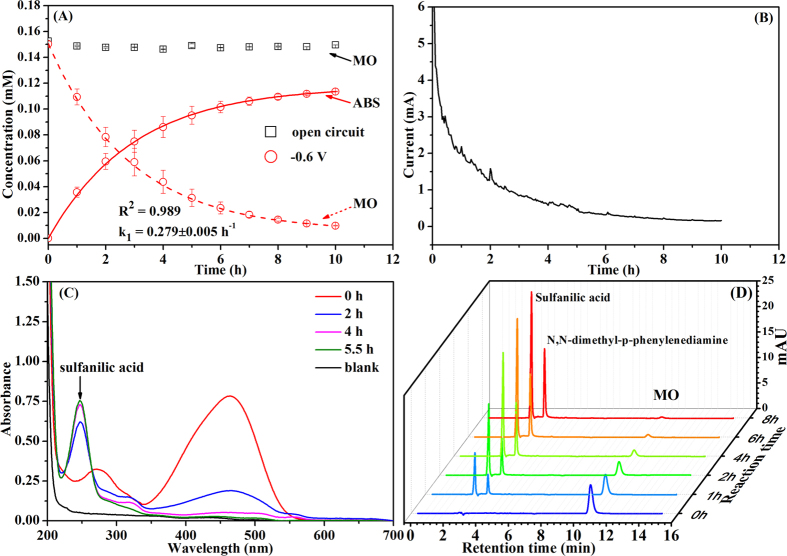
Typical experiment in the BES: (**A**) MO decolourization and ABS formation and (**B**) current generation at open and closed circuit with controlled cathode potential of −0.6 V respectively, (**C**) UV absorption spectra and (**D**) HPLC chromatogram during MO decolourizaiton at cathode potential of −0.6 V.

Figure 1A illustrates typical MO removal in the BES at open and closed circuits, respectively. The MO concentration varied slightly with reaction time in the open circuit, indicating insignificant MO adsorption on the reactor or electrode materials. When cathode potential was controlled at −0.6 V vs Ag/AgCl (all potentials provided refer to Ag/AgCl reference electrode in this study), MO concentration was rapidly reduced to almost zero after 10-h operation in the BES. Moreover, the MO decolourization in the BES was well fit to the pseudo-first kinetic model (*R*^*2*^ = 0.989) with the rate constant (*k*_*1*_) of 0.279 ± 0.005 h^−1^. Additionally, the BES current was gradually reduced from 11.73 ± 5.82 to 0.18 ± 0.04 mA during MO decolourization (Fig. 1B).

The successful degradation of the azo dye was proved through UV-vis absorption spectra of the reaction solutions. As shown in Fig. 1C, the absorbance band at 465 nm, which originated from a conjugated structure formed by the azo bond^39^, decreased and shifted to a longer wavelength after 2-h reduction, whereas an absorbance band at 247 nm was formed, which was possibly ascribed to sulfanilic acid (ABS)^40^. With increased reaction time to 4 and 5.5 h, the band at 465 nm continuously decreased, which is contrary for the band at 247 nm. The evolution of the absorbance spectra in Fig. 1C demonstrates the cleavage of the azo bond and the formation of reductive products. When azo dye was reduced, azo double bond was destroyed and the absorbance caused by the azo group (at 465 nm) became lower, while the increased absorbance at 247 nm resulted from the aromatic parts of the molecule. Furthermore, the reductive products of MO decolourization were identified through HPLC chromatogram (Fig. 1D). The main peaks in the solution with retention times of 2.62 and 3.42 min matched exactly with those of ABS and N, N-dimethyl-p-phenylenediamine respectively, indicating that these two compounds were the dominant reductive products of MO decolourization in the BES. As shown in Fig. 1A, the concentration of ABS, one of reductive products, was increased from zero to 0.11 ± 2.17 mM with reactor time during MO decolourization. However, another product of MO decolourization, N, N-dimethyl-p-phenylenediamine, was hardly quantified because it is unstable and can easily autoxidized even in the presence of small amount of oxygen in the solution^41^.

### Effect of cathodic potential

**Figure 2:**
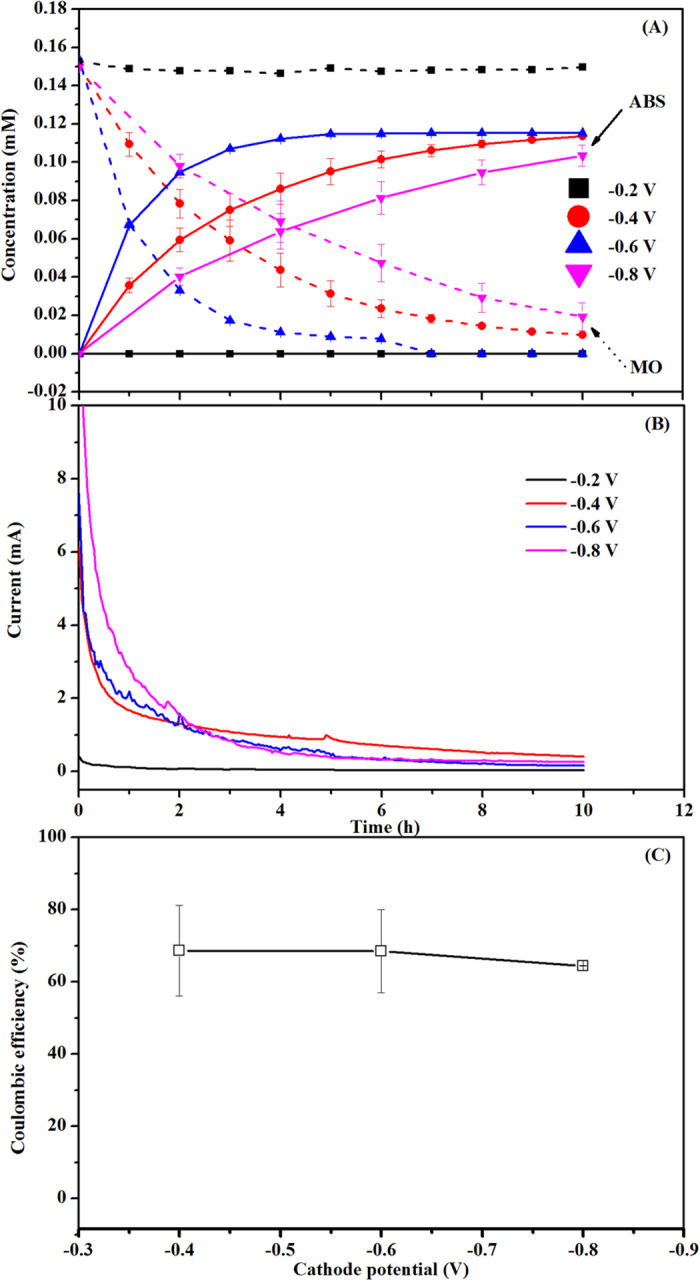
Effect of cathode potential on (**A**) MO decolourization and ABS formation, (**B**) current generation, and (**C**) Coulombic efficiency.

Figure 2A illustrates MO removal at different cathode potentials in the BES. MO decolourization was neglectable at the controlled cathode potential of −0.2 V and the current was almost zero (Fig. 2B), but it was remarkably enhanced when the cathode potential was changed to −0.4, −0.6 and −0.8 V in the BES. The MO decolourization efficiency after 10 h increased from 0 to 94.90 ± 0.01% with decreasing cathode potential from −0.2 to −0.8 V. Correspondingly, ABS formation was also enhanced in the BESs (Fig. 2A). Additionally, the BES current was decreased with reaction time during MO decolourization under different cathode potentials (Fig. 2B). Lower cathode potential resulted in higher current generation especially at the beginning of reaction in the BES. The Coulombic efficiency (CE) of the MO removal at the cathode was evaluated as the ratio of the current that would be obtained as a result of the complete breakdown of the MO azo bond and the current flowing across the BES. As shown in Fig. 2C, the CE for MO decolourization slightly varied from 64.43 ± 0.06% to 68.63 ± 12.53% with the cathode potential in the range of −0.4 to −0.8 V, indicating that a majority of electrons from cathode electrode were used for MO decolourization, i.e., azo bond breakdown at the cathode. As shown in Table 1, the correlation coefficients *R*^2^ were higher than 0.94, suggesting that MO decolourization at various cathode potentials could be well described with the pseudo-first order kinetic model in the BES. When cathode potential was reduced from −0.2 to −0.8 V, the reaction rate constant was increased from 0 to 0.503 ± 0.001 h^−1^. Similarly, Mu *et al.* observed that decolourization efficiency of azo dye Acid Orange 7 increased from 70.90 ± 2.60% to 98.70 ± 2.50% by controlling the cathode potential in the range of −0.15 to −0.35 V in the BES^16^. Sun *et al.* reported the decolourization activities of azo dye Alizarin Yellow R were significantly enhanced as cathode potential decreased from −0.6 to −1.2 V in the BES, supplying either acetate or glucose as co-substrates^29^. Cui *et al.* also found that decolourization efficiency of azo dye Alizarin Yellow R was improved when cathode potential of the BES decreased^23^. A more negative cathode potential means a better reduced environment that may be more beneficial to electron transfer from electrode to azo dye for its reduction in the BES.

### Effect of DO concentration in the cathode chamber

**Figure 3:**
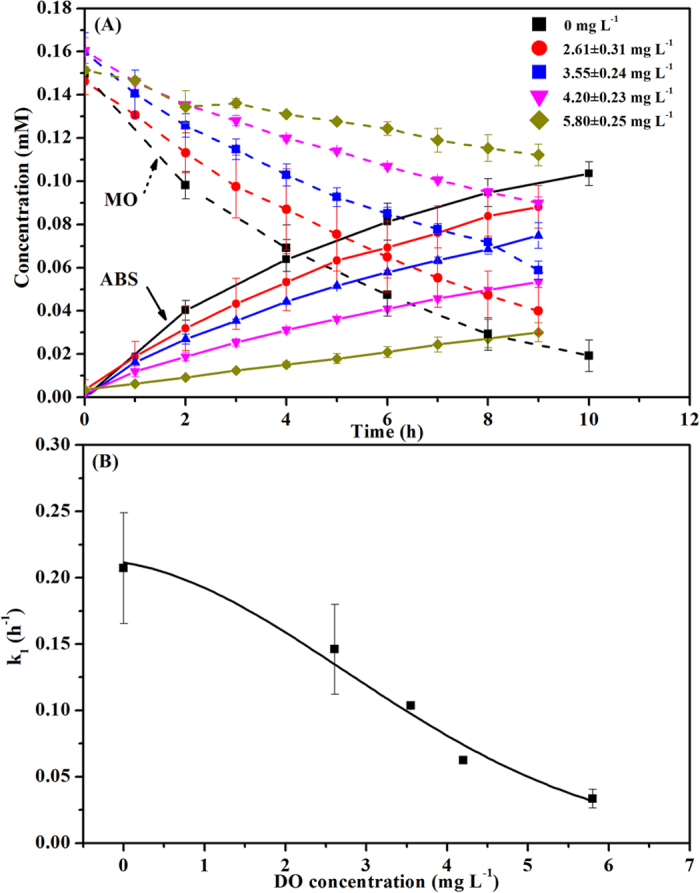
Effect of DO concentration in the cathode chamber on (**A**) MO decolourization and ABS formation, and (**B**) pseudo first-order kinetic rate constant.

The results of MO decolourization under different DO concentrations of catholyte (2.61 ± 0.31, 3.55 ± 0.24, 4.20 ± 0.23, 5.80 ± 0.25 mg L^−1^) in the BES are shown in Fig. 3. MO decolourization efficiency over 9-h reaction decreased from 87.19 ± 4.73% to 27.77 ± 0.06% with the increase of DO concentration from 0 to 5.80 ± 0.25 mg L^−1^ at a controlled cathode potential of −0.4 V in the BES. Correspondingly, the ABS concentration reduced from 0.103 ± 0.006 to 0.030 ± 0.004 mM. Under different DO concentrations in the cathode chamber of the BES, MO decolourization also followed the pseudo-first order kinetic reaction with correlation coefficients *R*^2^ higher than 0.99. As shown in Fig. 3B, the reaction rate constant of MO removal was reduced from 0.207 ± 0.042 to 0.033 ± 0.007 h^−1^ with increased DO concentration from zero to 5.80 ± 0.25 mg L^−1^ in the cathode chamber. These results indicate a negative effect of DO in the cathode chamber on the MO decolourization, which might be due to that O_2_ is the most plausible sink of electrons and could compete for electrons from the cathode with azo dye in the BES^22^. Virdis *et al.* also found that DO could affect nitrification, denitrification and carbon removal in the cathode of the BES. Levels between 1.97 ± 0.09 and 4.35 ± 0.08 mg L^−1^ were not sufficient to significantly inhibit denitrification, while under conditions of higher aeration, i.e., 5.02 ± 0.02 to 7.24 ± 0.10 mg L^−1^ DO, denitrificaton was seriously limited^42^.

### Effect of cathodic biofilm

**Figure 4:**
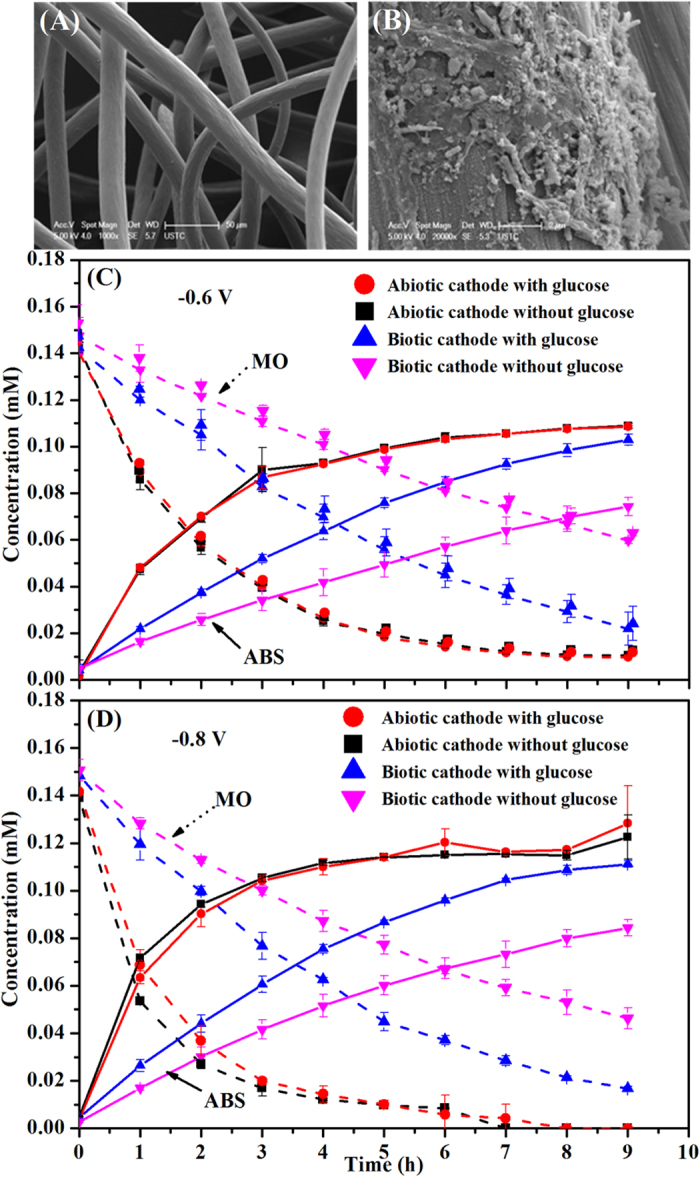
SEM image of cathode (**A**) without biofilm and (**B**) with biofilm, MO decolourization and ABS formation with and without biofilm on the cathode at controlled cathode potential (**C**) −0.6 V and (**D**) −0.8 V, respectively.

**Figure 5:**
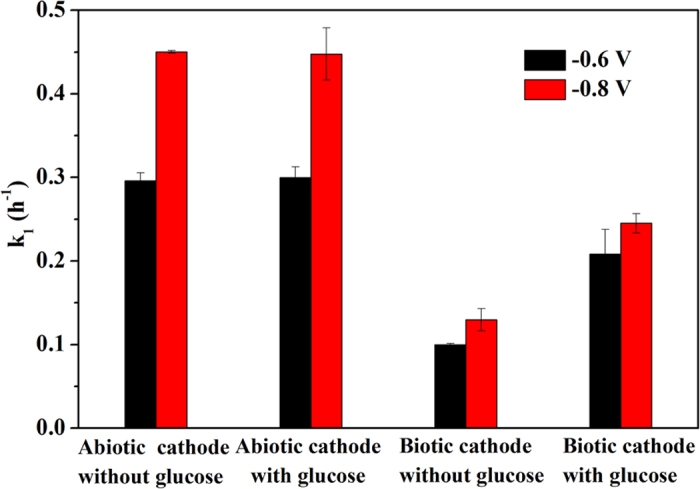
Comparison of pseudo first-order kinetic rate constant of MO decolourization with and without biofilm on the cathode in the BES.

SEM images reveal that a biofilm was formed on the surface of the cathode (Fig. 4A), while the abiotic cathode was smooth without microorganism attachment (Fig. 4B). The effect of cathodic biofilm on MO decolourization and ABS formation in the BES is presented in Fig. 4C,D, respectively. Without biofilm formed on the cathode, MO decolourization efficiency was about 92.61 ± 0.65% at a controlled cathode potential of −0.6 V (Fig. 4C), moreover, the presence of glucose in the cathode chamber of the BES almost had no effect on MO decolourization efficiency. However, MO decolourization efficiency was significantly reduced to 59.44 ± 1.31% when biofilm was formed on the cathode in the BES, while addition of glucose in the cathode resulted in a higher MO decolourization efficiency of 84.56 ± 5.20%. As a result, the formation of ABS was negatively impacted in the presence of biofilm on the cathode in the BES, but could be enhanced through dosing glucose into the cathode. The similar results were also obtained at a controlled cathode potential of −0.8 V, as shown in Fig. 4D. MO decolourization in the BES still followed the pseudo-first order kinetic reaction in the present of biofilm on the cathode. As shown in Fig. 5, without biofilm formed on the cathode, the MO decolourization rate constant was similar in the presence and absence of glucose at two controlled cathode potentials in the BES. However, the MO decolourization reaction rate constant had a substantial reduction when biofilm was formed on the cathode. Above results suggest a negative rather than a positive influence of cathodic biofilm on MO decolourization in the BES, which might be due to that biofilm took up some reaction sites on the cathode and thus prevented electron transfer from cathode to azo dye. On the other hand, the addition of glucose in the cathode chamber could substantially enhance MO decolourization in the BES with biotic cathode, implying that the some microorganisms in the biofilm formed on the cathode were likely able to use glucose as the electron donor for MO decolourization. Most of previous studies reported that the biofilm formed on the cathode could positively affect the reduction of contaminants^26^,^27^,^28^,^29^,^30^,^31^. For instance, pentachlorophenol degradation rate were improved by 21.50% in the biocathode of MFCs^30^. 88.20 ± 0.60% of the nitrobenzene was transformed to aniline within 24 h using a fed-batch BES with microbially catalyzed cathode, which was higher than an abiotic cathode^35^. In addition, Kong *et al.* reported that decolourization of azo dye Congo red was accelerated in a combined bioanode-biocathode BES^25^. However, our results were contrary to those studies, suggesting that some other types of microorganisms rather than the electrochemically active bacteria could be potentially enriched on the cathode to negatively influence azo dye decolourzation.

## Materials and Methods

### Reactor

**Figure 6:**
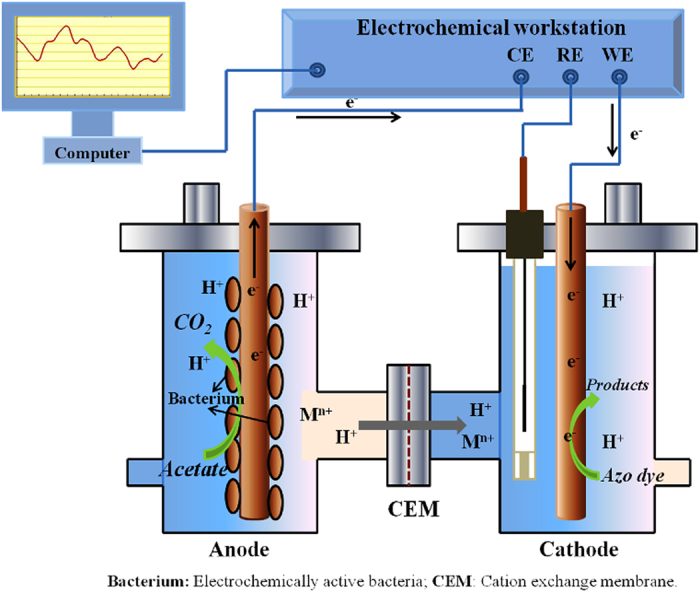
Schematic diagram of the BES.

As shown in Fig. 6, BESs were constructed according to a previous report^43^. The anode and the cathode each in a 450-mL chamber were separated by a cation exchange membrane (Membranes International Inc., USA). The anode was filled with graphite granules (average diameter 0.2–0.6 cm), making the net volume of anode 200 mL, and carbon felt (3.5 × 5.0 cm) was used as cathode material. An Ag/AgCl reference electrode was placed close to the cathode electrode for potential measurement. The closed circulation was connected in series with an electrochemical workstation (Bio-Logic Science Instruments, France) for online measurement and control.

During reactor startup, the anodic compartment of the BES was inoculated with anaerobic sludge and was fed into acetate as substrate. The anode chamber was connected with a 1-L bottle containing the growth medium^22^, and was recirculated by the peristaltic pump at an approximate rate of 50 mL min^−1^. The growth medium of 1-L solution contained: KCl, 52 mg; CaCl_2_, 10 mg; MgCl_2_·6H_2_O, 72 mg; FeSO_4_·7H_2_O, 3.2 mg; CoCl_2_·2H_2_O, 1 mg; MnCl_2_·4H_2_O, 0.8 mg; Na_2_Mo_7_O_4_·2H_2_O, 3 mg; H_3_BO_3_, 0.2 mg; NiCl_2_·6H_2_O, 0.5 mg; CuCl_2_·2H_2_O, 1.1 mg; ZnSO_4_·7H_2_O, 3.2 mg; EDTA, 1 mg; NH_4_Cl, 0.31 g. The growth medium contained 50 mM phosphate buffer (17.2 mM KH_2_PO_4_ and 32.8 mM Na_2_HPO_4_) to control anodic pH at 7.0. The open circuit potential of the anode was decreased to around −0.5 V after several months, indicating the stable biofilm had formed on the anode electrode in the reactor. 0.15 mM MO, close to dye concentration in real wastewaters^44^, was used as the electron acceptor in the cathode chamber of the BES, and 50 mM phosphate buffer was supplied to control pH at 7.0. To avoid acetate depletion in the anode chamber, anolyte was refreshed each time when the catholyte was renewed.

### Experiments

The effect of three key factors on MO decolourization in the BES was investigated and the experimental conditions are summarized in Table 2. Different cathode potentials from −0.2 to −0.8 V were controlled through the electrochemical workstation, while DO concentration in the cathode chamber was adjusted from 2.61 ± 0.31 to 5.80 ± 0.25 mg L^−1^ by varying flow rate of the continuous air aeration with a flow rotameter. During the experiments DO concentration of the catholyte was monitored with a DO meter (HQ 30d, Hach Co., USA). The cathodic biofilm was developed with anaerobic sludge as inoculum and glucose as substrate for four weeks. The inoculated anaerobic sludge was collected from a full-scale expanded granular sludge-bed reactor treating starch wastewater located in Shandong Province, China. During experiments, the pH of both anolyte and catholyte was controlled at 7.0 using phosphate buffer. Meanwhile, KCl was added into the both chambers to keep a constant ionic strength^45^. In addition, both anolyte and catholyte in the BES were sparged with nitrogen gas for at least 30 mins to remove dissolved oxygen before experiments except for DO investigation. In order to maintain well-mixed conditions and avoid concentration gradients, the cathode chamber of the BES was stirred with a magnetic stirrer. All experiments were conducted at least in duplicate and average values with standard deviation were presented in this study. And the BESs were maintained at room temperature throughout experimental period.

### Analysis and calculation

Throughout the experiments, samples taken from the BES were filtered immediately through a 0.22 μm membrane before analysis. The disappearance of MO was monitored by UV-vis spectrophotometer (Cary 60, Agilent Co., USA) over a wavelength range from 200 to 800 nm. MO and its reductive products were identified and quantified using high performance liquid chromatography (Model 1260, Agilent Co., USA) with HC-C18 column (reversed phase column, particle size 5 μm, 4.6 × 250 mm) and UV-detector (detection wavelength of 245 nm). Ammonium acetate (10 mM, pH 4.0) and methanol were used as the mobile phase with a volume ratio 1:1 at the flow rate of 0.5 ml min^−1^ 46.

MO decolourization in the BES was described by the pseudo-first order kinetic model:


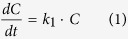


where *C* (mM) is the concentration of azo dye MO, *t* (h) is the reaction time, *k*_*1*_ (h^−1^) represents the apparent kinetic rate constant.

MO decolourization efficiency (DE, %) and Coulombic efficiency (CE, %) were calculated as follows^22^:


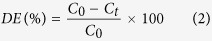


where *C*_0_ (mM) is the initial MO concentration, and *C*_*t*_ (mM) is the MO concentration at time t (h).


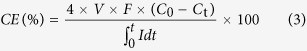


where 4 is the number moles of electrons that can be accepted by 1 mol of MO in the cathodic compartment assuming the complete breakdown of the azo bond, V (L) is the effective volume of the cathode chamber, F (96,485 C mol^−1^ e) is Faraday’s constant, and I (A) is the current.

## Additional Information

**How to cite this article**: Yang, H.-Y. *et al.* Process and kinetics of azo dye decolourization in bioelectrochemical systems: effect of several key factors. *Sci. Rep.* **6**, 27243; doi: 10.1038/srep27243 (2016).

## Figures and Tables

**Table 1:** Kinetics of MO decolourization under various cathode potentials.

Cathode potential (V)	Pseudo first-order kinetics	
	Rate constant *k*_1_ (h^−1^)	Correlation coefficients *R*^2^
−0.2	0	N.A.
−0.4	0.207 ± 0.042	0.994
−0.6	0.279 ± 0.005	0.989
−0.8	0.503 ± 0.001	0.936

N.A.: not available

**Table 2:** Summary of operational conditions for each experimental setup in the BES.

Experiment	Operational conditions	
	Variable item	Constant item
Effect of cathode potential	Cathode potential: −0.2, −0.4, −0.6, −0.8 V	Anodic pH: 7.0 Cathodic pH: 7.0
Effect of DO concentration in the cathode chamber	DO concentration: 0, 2.61 ± 0.31, 3.55 ± 0.24, 4.20 ± 0.23, 5.80 ± 0.25 mg L^−1^	Cathode potential: −0.4 V Anodic pH: 7.0 Cathodic pH: 7.0
Effect of biofilm on the cathode	Abiotic cathode without glucose. Abiotic cathode with 1.0 g L^−1^ glucose. Biotic cathode without glucose. Biotic cathode with 1.0 g L^−1^ glucose	Cathode potential: −0.6, −0.8 V Anodic pH: 7.0 Cathodic pH: 7.0
